# Micromechanical Finite Element Model Investigation of Cracking Behavior and Construction-Related Deficiencies in Asphalt Mixtures

**DOI:** 10.3390/ma18153426

**Published:** 2025-07-22

**Authors:** Liu Yang, Suwei Hou, Haibo Yu

**Affiliations:** 1 Zhengzhou Railway Vocational and Technical College, Zhengzhou 451460, China; 2 Beijing University of Civil Engineering and Architecture, Beijing 102600, China; housuwei@bucea.edu.cn (S.H.); 2108590124165@stu.bucea.edu.cn (H.Y.)

**Keywords:** fracture mechanics, finite element simulation, indirect tensile test (IDT), cohesive zone model (CZM), random aggregate generation

## Abstract

This study investigated the fracture behavior of asphalt mixtures under indirect tensile loading by comparing the performance of homogenized and micromechanical finite element (FEMs) models based on the cohesive zone model (CZM). Five asphalt mixture types were tested experimentally, and both models were calibrated and validated using load–displacement curves from indirect tensile tests (IDTs). The micromechanical model, incorporating random aggregate generation and three-phase material definition, exhibited significantly higher predictive accuracy (R^2^ = 0.86–0.98) than the homogenized model (R^2^ = 0.66–0.77). The validated micromechanical model was further applied to quantify the impact of construction-related deficiencies—namely, increased air voids, non-continuous gradation, and aggregate segregation. The simulation results showed that higher void content (from 4% to 10%) reduced peak load by up to 35% and increased localized stress concentrations by up to 40%. Discontinuous gradation and uneven aggregate distribution also led to premature crack initiation and more complex fracture paths. These findings demonstrated the value of micromechanical modeling for evaluating sensitivity to mix design and compaction quality, providing a foundation for performance-based asphalt mixture optimization and durability improvement.

## 1. Introduction

Cracking is one of the most common distresses in asphalt pavements, primarily resulting from the combined effects of traffic loading and environmental factors. The progressive propagation of cracks can significantly reduce the structural bearing capacity of the pavement, thereby shortening its service life. Therefore, investigation of the fracture damage mechanisms and crack evolution behavior of asphalt mixtures are of great significance for enhancing pavement cracking resistance and long-term durability. In recent years, finite element method (FEM)-based numerical simulation has emerged as a powerful approach for elucidating fracture mechanisms in asphalt materials, and among these methods, the cohesive zone model (CZM) has gained widespread attention for its effectiveness in capturing crack initiation and propagation behavior [1,2,3]. Numerous studies have demonstrated that integrating numerical modeling with experimental methods—such as the single-edge notched beam (SENB), semi-circular bending (SCB), and indirect tensile test (IDT)—allows for the quantitative assessment of the effects of temperature, aggregate–binder interfacial properties, and aging conditions on fracture parameters [4,5,6,7].

Moreover, advances in microscale reconstruction techniques—enabled by CT scanning and digital image processing—have significantly improved the accuracy level of micromechanical models, enabling a deeper understanding of how aggregate distribution, air void content, and loading rate influence cracking resistance [8,9,10,11]. Despite these developments, many existing studies still rely on homogenized models, which assume asphalt mixtures as continuous and uniform materials. This simplification neglects the heterogeneous interactions among aggregates, mastic, and voids, thereby leading to substantial deviations between simulated load–displacement responses and experimental results [12,13]. Although the micromechanical FEMs have the theoretical capability to more accurately reflect the internal structure of asphalt mixtures [14,15], their practical application remains limited due to challenges such as stochastic aggregate generation algorithms, calibration of interfacial mechanical properties, and computational efficiency [9,16,17].

On the other hand, the long-term impact of common mixture deficiencies—such as inadequate compaction, gradation deviations, and uneven mixing—on mixture cracking performance has not been systematically quantified using FE models. Previous studies have shown that compaction procedures and aggregate morphology directly affect the contact characteristics of the aggregate skeleton [18,19,20], while gradation design significantly influences the dynamic modulus and damage evolution behavior [21,22]. However, most of the existing research focuses on the optimization of material constitutive models, with limited attention paid to the deterioration mechanism quantification of cracking resistance resulting from common construction issues. In particular, quantitative evaluation methods based on micromechanical FE modeling remain scarce [23,24]. Thus, the goal of this study was to increase the FE model computation performance and solve the mixture issues mentioned above.

To address the above gaps, this study presented two main contributions: (1) a micromechanical FE model integrating random aggregate generation and calibrated cohesive properties was constructed to systematically validate its predictive advantage over homogenized models using experimental IDT data, and (2) the validated micromechanical model was further applied to quantitatively assess the impact of three common construction deficiencies—void content variation, gradation deviation, and aggregate segregation—on cracking performance, which remained rarely reported in prior the literature. These contributions provided both theoretical innovation and practical relevance for improving asphalt mixture durability and quality control.

This paper was organized as follows. The asphalt mixture design and performance tests (i.e., dynamic modulus test and IDT) were presented in the next section. The third section included the methodology of the CZM and FEM. The simulation results were discussed in the fourth section, followed by the application using the mesoscopic model to evaluate the common construction deficiencies. The last section concluded the summary and future work.

## 2. Materials and Methods

### 2.1. Test Materials and Mix Parameters

#### Material Parameters

This study utilized five types of asphalt mixtures. The asphalt content, Reclaimed Asphalt Pavement (RAP) content, air voids, and mix design details for each mixture type are presented in Table 1 and Table 2, respectively.

### 2.2. Equipment and Testing Conditions

#### 2.2.1. Dynamic Modulus Test

To comprehensively evaluate the linear viscoelastic behavior of asphalt mixtures under varying loading frequencies and temperature conditions, dynamic modulus tests were carried out on five asphalt mixture types with distinct mix designs. These experimental results served as the basis for constructing the master curves, which were subsequently transformed into relaxation moduli. This conversion facilitates a precise definition of the time-domain viscoelastic constitutive relationship for asphalt mixtures when implemented in the finite element program ABAQUS (Version 2021, Dassault Systèmes, Ile-de-France, France).

The test specimens were fabricated using the Superpave Gyratory Compactor to simulate field compaction conditions and then cut into standard cylindrical specimens with a nominal diameter of 100 mm and a height of 170 mm. To ensure statistical reliability and repeatability of the test results, no fewer than three replicate specimens were prepared for each mixture type. The dynamic modulus tests were performed under axial stress-controlled loading using a sinusoidal waveform at a single frequency per test. The loading frequencies covered six representative levels: 0.1 Hz, 0.5 Hz, 1 Hz, 5 Hz, 10 Hz, and 25 Hz. For each frequency level, a minimum of ten loading cycles were applied, and only the stabilized (steady-state) segments of the stress–strain response were used for subsequent analysis.

Testing was conducted at five temperature levels, −10 °C, 4.4 °C, 21.1 °C, 37.8 °C, and 54.4 °C, to simulate a wide range of field service conditions. Prior to testing, all specimens were thermally conditioned in a controlled environmental chamber for at least two hours to ensure uniform internal temperature and thermal equilibrium. To unify the viscoelastic response data across different temperatures and loading frequencies, the time–temperature superposition principle was employed to shift the data horizontally and construct a master curve at reference temperature of 21.1 °C. The temperature shift factor $\alpha_{T}$ was fitted using the Williams–Landel–Ferry (WLF) equation, as shown in Equations (1) and (2), respectively.

(1) $$E(f_{r})=\delta +\frac{\alpha }{(1+\lambda e^{\beta +\gamma logf_{R}}{)}^{\frac{1}{\lambda }}}$$

(2) $$\alpha_{T}=10^{\frac{-C_{1}\left(T-T_{R}\right)}{C_{2}+\left(T-T_{R}\right)}}$$

In Equations (1) and (2), the parameters α, β, γ, λ, and δ are dimensionless model fitting constants that define the shape of the dynamic modulus master curve. $f_{r}$ is the reduced frequency, expressed in hertz (Hz). The WLF equation is used to calculate the temperature shift factor $\alpha_{T}$, which is dimensionless. In this equation, $C_{1}$ and $C_{2}$ are dimensionless empirical constants obtained by curve fitting. T is the test temperature, and $T_{R}$ is the reference temperature, both expressed in degrees Celsius (°C). These parameters together enable the construction of a time–temperature superposition master curve for the dynamic modulus.

**Figure 1:**
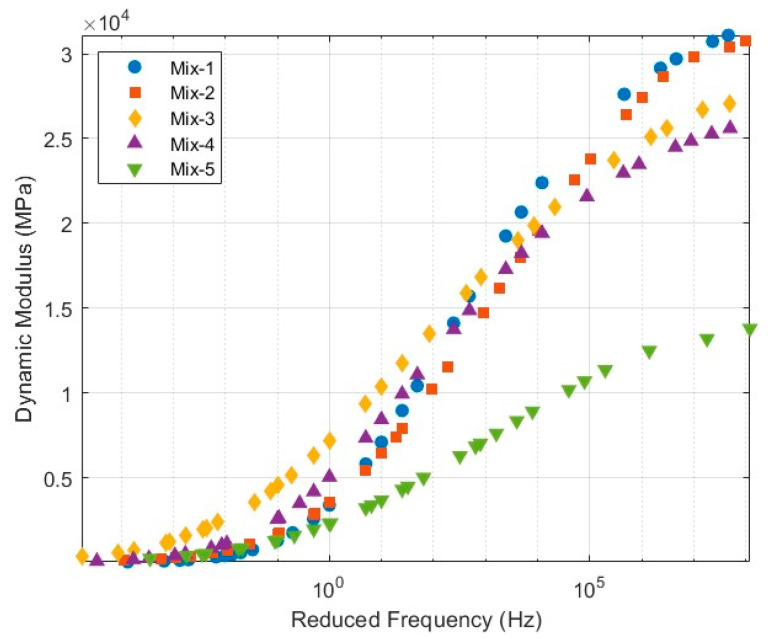
Master curves of dynamic modulus for five asphalt mixtures.

The temperature–frequency data were shifted horizontally to a reference temperature using the time–temperature superposition principle, resulting in a continuous master curve, as illustrated in Figure 1. This master curve serves as the basis for subsequent viscoelastic constitutive modeling.

To define the viscoelastic behavior in ABAQUS, the dynamic modulus master curve must be transformed into a relaxation modulus function in the time domain using the Prony series representation. The corresponding calculation formulas are given in Equations (3)–(6). The Prony series parameters were determined through nonlinear least squares fitting.

(3) $$E^{\prime }(\omega )=E_{\infty }+\sum_{j=1}^{M}\frac{\omega^{2}j_{2}^{k}E_{j}}{1+\omega^{2}j_{2}^{k}}$$

(4) $$E^{\prime \prime }(\omega )=\sum_{j=1}^{M}\frac{\omega k_{j}E_{j}}{1+\omega^{2}j_{2}^{k}}$$

(5) $$E(t)=E_{\infty }+\sum_{j=1}^{M}E_{j}exp\left(-\frac{t}{k_{j}}\right)$$

(6) $$E(0)=E_{\infty }+\sum_{j=1}^{M}E_{i}$$

The dynamic modulus master curve needs be transformed into a relaxation modulus function in the time domain using the Prony series representation. The corresponding equations are given in Equations (3)–(6). $E^{\prime }\left(\omega \right)$ and $E^{\prime \prime }\left(\omega \right)$ represent the storage modulus and loss modulus, respectively, both expressed in megapascals (MPa). These describe the elastic and viscous components of the complex modulus at a given angular frequency ω, where ω = 2πf and f is the loading frequency in hertz (Hz).

The relaxation modulus $E\left(t\right)$ (in MPa) describes the material’s stress relaxation over time under a constant strain. $E\left(0\right)$ is the instantaneous modulus (MPa), reflecting the material stiffness at the moment of loading, while $E_{\infty }$ is the long-term equilibrium modulus (MPa), representing the residual stiffness after complete relaxation.

$E_{j}$ corresponds to the individual components of the relaxation modulus, while $k_{j}$ denotes the associated relaxation times. These parameters together define the viscoelastic behavior of asphalt mixtures in the finite element model.

#### 2.2.2. IDT Test

**Figure 2:**
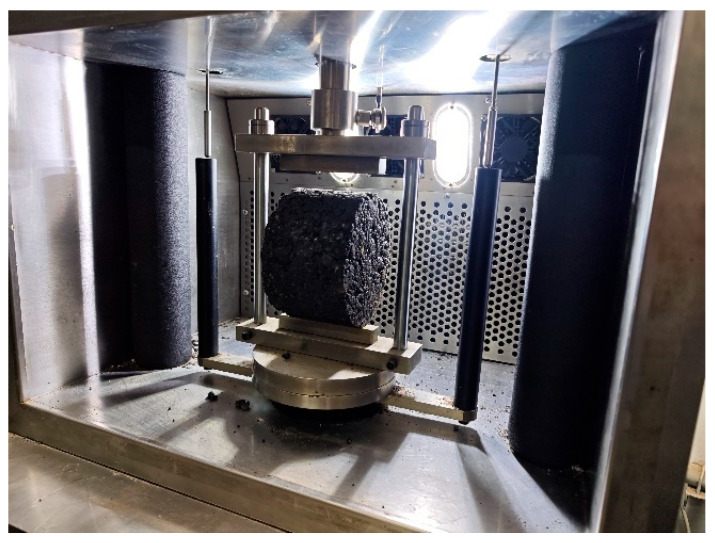
IDT apparatus.

The fracture resistance of five distinct types of plant-produced asphalt mixtures were evaluated through the IDT. Prior to compaction, the loose mixtures underwent short-term oven aging at 135 °C for a duration of two hours, simulating the aging that typically occurs during production and laydown. Compaction was carried out using a Superpave Gyratory Compactor to achieve the target density and simulate field compaction conditions. The IDT specimens were fabricated with a nominal diameter of 150 mm and a thickness of 63.5 mm in accordance with standard testing specifications. To ensure data reliability and repeatability, a minimum of three replicate specimens were prepared for each asphalt mixture type. The IDT was conducted using a universal asphalt mixture testing machine equipped with a customized loading fixture. A vertical load was applied to induce radial tensile strain in the specimen. The test automatically terminated when the specimen fractured into two pieces. The test setup is illustrated in Figure 2.

The loading head was cylindrical, and the contact surface between the loading head and the specimen was curved. The radius of curvature of the loading head was designed to match that of the specimen to minimize stress concentration at the contact interface. The test was performed under displacement-controlled mode. The loading rate was set at 50 mm/min. The temperature within the environmental chamber was maintained at a constant ambient temperature of 25 °C. Throughout the entire test, load and displacement data were recorded at 0.02 s intervals.

## 3. Numerical Simulation Methods and FEM Development

### 3.1. Theoretical Basis and Fundamental Assumptions

#### 3.1.1. Introduction of CZM

**Figure 3:**
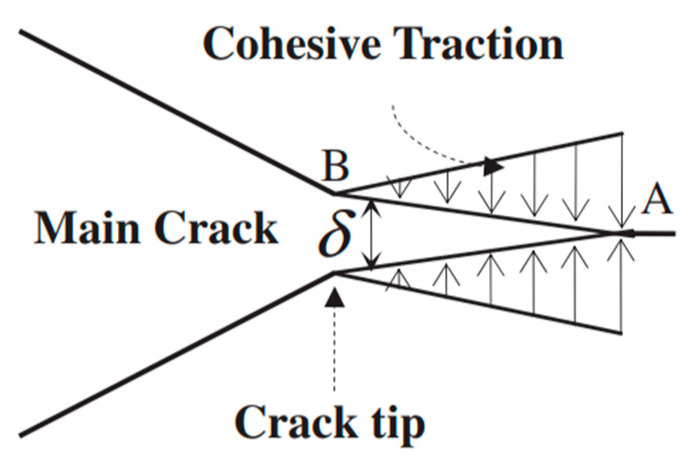
Schematic diagram of the cohesive zone.

**Figure 4:**
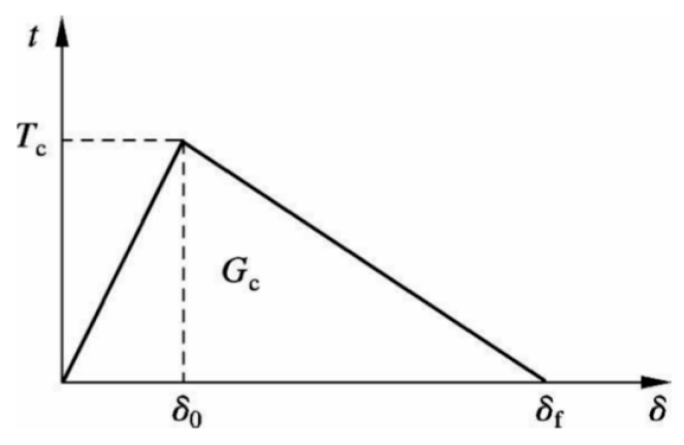
Bilinear cohesive force constitutive model.

The bilinear CZM is a widely used method for describing material fracture and interface failure, with its theoretical foundation based on fracture mechanics and energy dissipation principles, which is illustrated in Figure 3. The constitutive relationship of the bilinear CZM is shown in Figure 4. Here, t represents the cohesive stress; δ is the relative displacement of the fracture surface; T_c_ is the tensile strength of the material; δ_0_ is the displacement at which the cohesive stress reaches T; δ_f_ is the maximum displacement of the crack surface, referred to as the failure displacement; and G_c_ is the fracture energy. The bilinear CZM assumes the following: before the cohesive stress reaches the fracture strength (δ < δ_0_), the material in the cohesive zone behaves elastically; after the stress reaches the fracture strength (δ > δ_0_), the material exhibits linear softening behavior. The interval δ_0_ < δ < δ_f_ represents the softening phase, also referred to as the damage phase. A typical bilinear CZM is characterized by three main parameters: interface stiffness K is expressed in units of MPa/mm, tensile strength T_c_ is expressed in MPa, and fracture energy G_c_ is measured in J/m^2^. The relationship between the interface stiffness K, tensile strength T_c_, and fracture energy G_c_ is expressed by Equation (7).

(7) $$G_{c}=\frac{1}{2}T_{c}\delta_{f}$$

**Figure 5:**
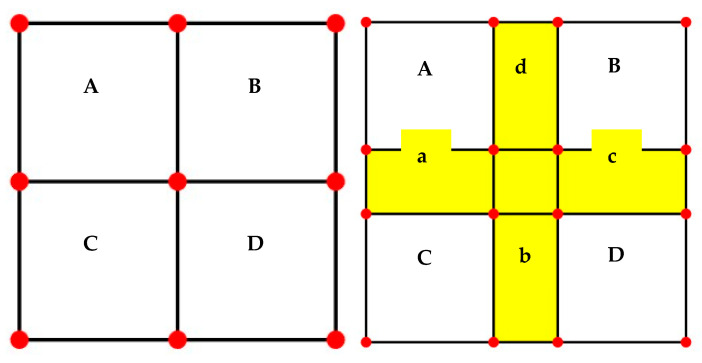
Insertion location of zero-thickness cohesive elements.

In this study, the bilinear CZM was implemented via cohesive elements within the finite element software ABAQUS. To enable free crack propagation within the asphalt concrete system, cohesive elements were embedded along the internal interfaces of different material phases. The insertion strategy adopted for the zero-thickness cohesive elements is illustrated in Figure 5. The model input file (.inp) was generated in ABAQUS, and the corresponding node and element identifiers were defined. Specifically, cohesive elements a, b, c, and d were introduced along the interfaces AC, CD, DB, and AB, respectively, thereby forming zero-thickness cohesive zones that accurately simulate interfacial debonding behavior.

#### 3.1.2. Random Aggregate and Void Generation Theory

**Figure 6:**
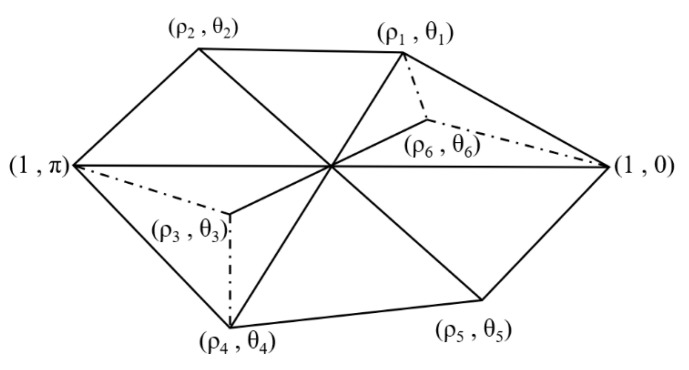
Schematic diagram of random aggregate and void generation.

In view of the random distribution characteristics of aggregates and voids in asphalt mixtures, Castillo et al. [25] proposed a random placement algorithm to generate two-dimensional virtual asphalt mixture specimens. This study further employed viscoelastic damage constitutive modeling to analyze the effect of microstructure on the development of damage within the asphalt mixture. A modeling approach based on random aggregate generation was adopted. The model was constructed and meshed using the ABAQUS software, and the corresponding .inp file was utilized to extract essential information, including node numbers, element connectivity, and boundary conditions, to facilitate the random aggregate generation process. Polygonal aggregates with irregular shapes were generated using polar coordinates, defined by randomly assigned angles and radial distances. The vertex coordinates of these random polygons were subsequently transformed into the global coordinate system of the FEM, as illustrated in Figure 6. Aggregates of each size category were sequentially introduced. Once the total area of successfully placed aggregates met the target content defined by the two-dimensional gradation curve, the generation of the next aggregate size was commenced. This iterative process continued until the cumulative area of aggregates in all size fractions satisfied the specified gradation. To simulate the presence of air voids, elements within the asphalt binder matrix were randomly deleted according to the designated porosity. Upon completion of this step, the program was terminated. As a result, a finite element meso-structure model comprising asphalt binder, coarse aggregates, and air voids was successfully established.

### 3.2. Homogeneous Model Development

**Figure 7:**
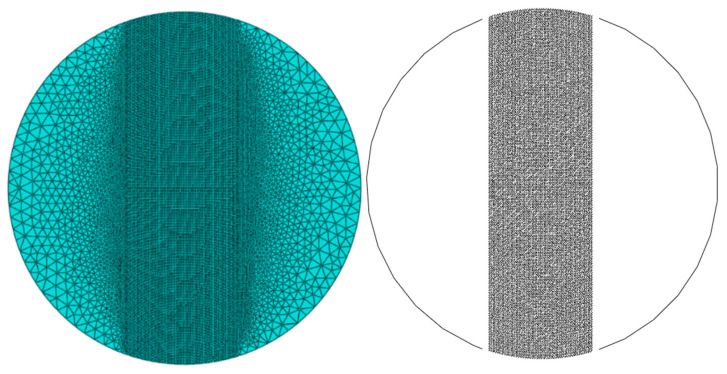
Arrangement of the homogeneous model and cohesion elements.

Based on the experimental parameters and modeling methods, a homogeneous model for the IDT specimen was established, with a model diameter of 150 mm and an out-of-plane thickness of 63.5 mm, consistent with the real specimen. The asphalt mixture was treated as a continuous medium, without considering the aggregate gradation and void distribution. The material property parameters are shown in Table 3. A three-node linear plane-strain triangular element (CPE3) was used, while four-node two-dimensional cohesive elements (COH2D4) were inserted in the central region where cracks were likely to initiate and propagate. In contrast, no cohesive elements were introduced in the edge regions where crack initiation and propagation were less probable. To ensure simulation accuracy while reducing the number of cohesive elements, computational resource consumption was minimized to balance computational efficiency and model reliability effectively. A mesh size of 1 mm was selected for the central region, while a 5 mm mesh size was used for the edge region, with a gradual transition towards the central region. Figure 7 illustrates the mesh division and the placement of cohesive elements in the homogeneous model of the IDT specimen.

Although the IDT specimen has a relatively small thickness (63.5 mm), a plane strain assumption was adopted in this study to ensure numerical stability and better simulate mid-plane crack propagation in the 2D FEM framework [26]. Previous studies have also employed plane strain elements for IDT simulations, as the cohesive zone modeling often focuses on the central cross-section, where out-of-plane deformations are limited. Moreover, comparative tests performed in this study showed that plane strain simulations yield nearly identical load–displacement responses compared to plane stress, while providing more consistent crack path predictions and avoiding convergence issues during cohesive element softening. Therefore, the plane strain formulation was considered a reasonable and effective simplification.

In the homogeneous model, the asphalt mixture was assumed to behave as an isotropic linear elastic material. The elastic modulus and Poisson’s ratio were determined based on experimental calibration, as listed in Table 3. No damage or viscoelasticity was considered in the bulk material, and fracture behavior was solely represented by cohesive zone elements inserted in the central region.

To replicate the IDT conditions in the FEM, appropriate boundary constraints and loading schemes were defined. The numerical specimen had the same dimensions as the physical sample (150 mm in diameter and 63.5 mm in thickness in 2D plane). The lower semicircle of the specimen was fully constrained in both the horizontal and vertical directions, while the upper semicircle was allowed to move only in the vertical direction. A vertical displacement-controlled load was applied through the upper loading strip at a constant rate of 50 mm/min, consistent with the experimental setup.

To minimize stress concentrations and ensure smooth load transfer, a rounded loading head was modeled with a curvature radius matching the specimen’s surface, and contact interfaces were defined with appropriate friction coefficients. The loading was applied through a reference point coupled to the top loading surface using a multi-point constraint (MPC) approach. No lateral constraints were applied to the specimen edges to avoid artificial confinement.

The same loading and boundary condition strategy was used for both the homogeneous and micromechanical models to ensure comparability of simulation results.

### 3.3. Mesoscopic Model Development

#### 3.3.1. Division of Three-Phase Material Model

To realistically represent the internal structure and mechanical response of asphalt mixtures, the mixture was divided into three phases: coarse aggregates, asphalt mastic, and voids. Coarse aggregates, with particle sizes greater than 2.36 mm, served as the skeletal material and possessed mechanical properties significantly higher than those of the other phases. Asphalt mastic consisted of fine aggregates (particles smaller than 2.36 mm) and asphalt binder. Due to the small size and large quantity of fine aggregates, a continuum hypothesis was typically adopted. In the FEM, to simplify the model and calculations, voids in the asphalt mixture were often idealized as uniformly distributed circular holes. This approach is commonly used in the analysis of overall mechanical responses, as the circular shape facilitates meshing. The diameter of these circular voids can be calculated using a regression formula [27], with specific values provided in Table 4.

(8) $$c=0.0037(\%AV{)}^{2}+0.0071(\%AV)+0.5583\;(R^{2}=0.7431)$$

**Figure 8:**
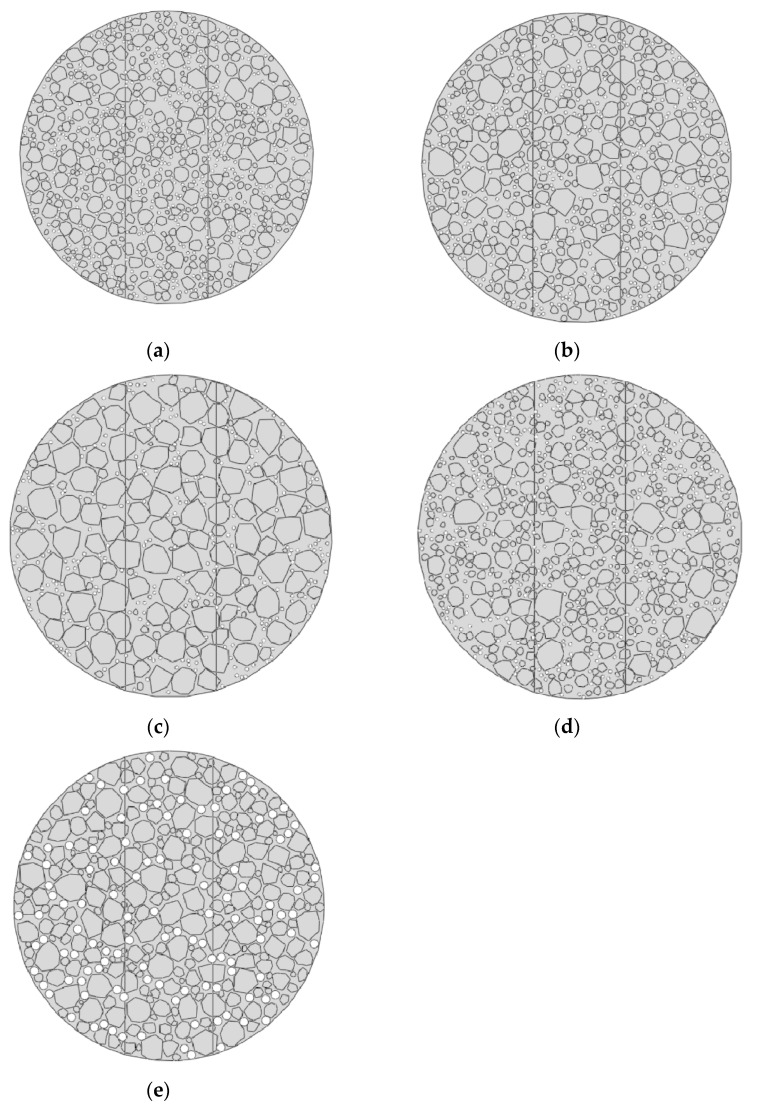
Finite element micromechanical models of five asphalt mixtures: (**a**) Mix-1, (**b**) Mix-2, (**c**) Mix-3, (**d**) Mix-4, and (**e**) Mix-5.

The theory and methods for generating random aggregates and voids are detailed in Section 3.1.2 and were not repeated here. For each material, the modeling was performed based on its gradation information, as shown in Figure 8. The two vertical lines in the model were set to distinguish the central region, where cracks may initiate and propagate. Note that these lines did not affect the computational results or accuracy.

#### 3.3.2. Placement of Cohesive Elements in Each Phase

To simulate the cohesive failure and crack propagation within and at the interfaces of each phase, cohesive elements were arranged in the following three regions of the mesoscopic model:

- Coarse Aggregate Region: The coarse aggregate was relatively stiff overall. Therefore, the cohesive element parameters in this region were set higher to reflect its high load-bearing capacity and relatively low ductility.
- Asphalt Mastic Region: Due to the material properties of asphalt mastic, it exhibited pronounced nonlinear behavior. The cohesive elements in this region were designed to reflect lower initial stiffness, higher cracking resistance, and higher plastic deformation possibility.
- Interface Region (Aggregate–Mastic Interface): The interface between the aggregate and mastic represented a weak link in stress transfer within the mixture, prone to interface debonding or crack propagation. The cohesive elements in this region exhibited significantly reduced interface stiffness and fracture toughness compared to the interior of the other two phases.

**Figure 9:**
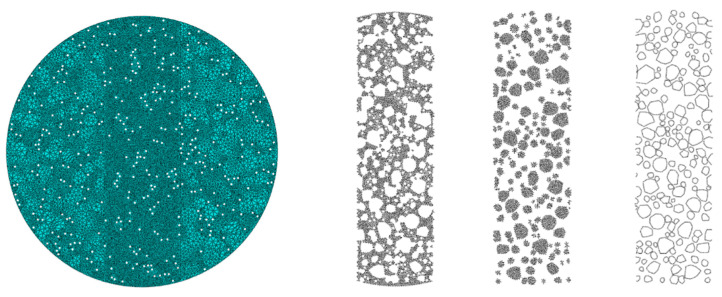
Micromechanical model and arrangement of cohesion elements.

The mesh division and arrangement used were similar to those in the homogeneous model. Figure 9 illustrates the mesh division and the placement of cohesive elements in Mix-1 IDT specimen’s mesoscopic model. The mesoscopic models of other materials were similar to this model.

The material property parameters for the three-phase model are shown in Table 5. Based on the previous research and a comprehensive consideration of the parameter ranges for rock materials (40,000 MPa, 60,000 MPa), the tensile strength and the fracture energy were taken as 90% and 50% of the asphalt mastic strength and of the asphalt mastic strength, respectively [28], considering the strength degradation at the interface. Asphalt mortar was modeled as a linear viscoelastic material using a Prony series representation, based on experimental dynamic modulus test data. The time-domain relaxation modulus was obtained by fitting the master curve using the WLF shift factors and converted for ABAQUS implementation. This allowed for the accurate simulation of rate-dependent deformation in the binder-rich phase.

The mesoscopic parameters can be obtained through the indirect conversion of macroscopic parameters from the laboratory tests or by referring to existing research results. However, regardless of the method used to obtain the parameters, they may not necessarily align perfectly with the model. Therefore, it is necessary to continuously adjust the mesoscopic parameters, perform iterative trial simulations, and compare the results with laboratory test outcomes to determine the final model parameters that best fit the actual conditions. This process was also referred to as parameter calibration.

The mesoscopic model maintained the same loading conditions as the homogeneous model, while more detailed convergence controls and numerical stability analyses were implemented to address the complexity of the mesoscopic model.

## 4. Simulation Results and Quantitative Evaluation

### 4.1. Comparative Analysis of Load–Displacement Curves

**Figure 10:**
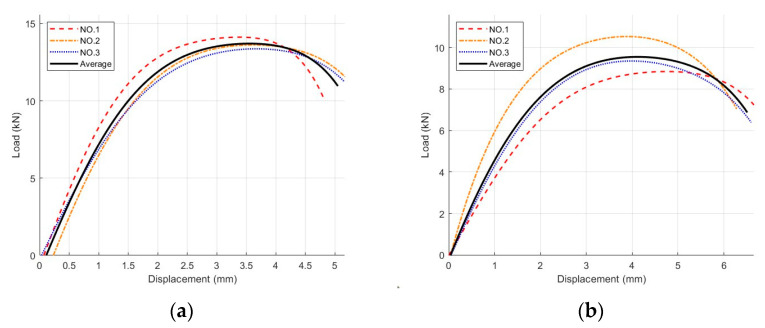

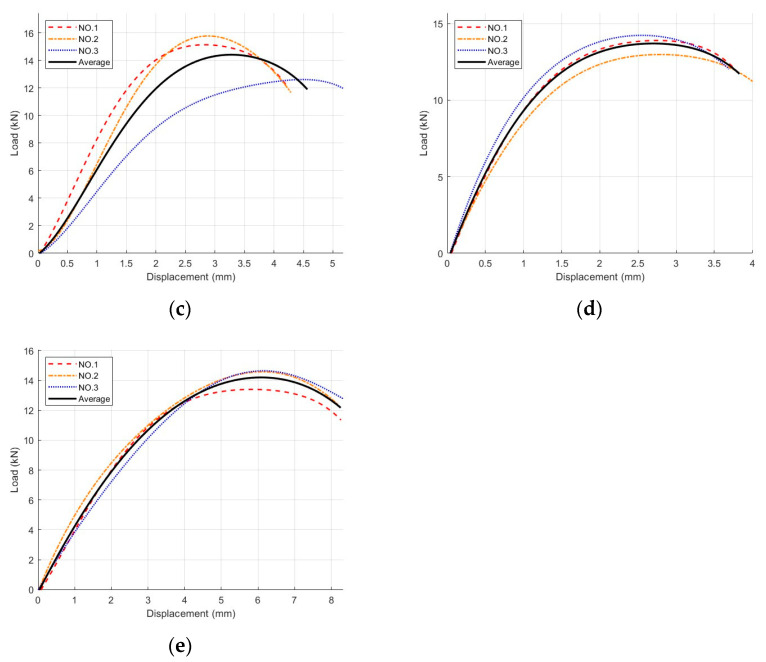
IDT load–displacement curves for five asphalt mixtures: (**a**) Mix-1, (**b**) Mix-2, (**c**) Mix-3, (**d**) Mix-4, and (**e**) Mix-5.

According to the experimental method described in Section 2.2.2, three replicate samples of each asphalt mixture were tested in the IDT, and their corresponding load–displacement curves are shown in Figure 10. The average values of the three load–displacement curves corresponding to the replicate samples were determined to produce a single load–displacement curve.

**Figure 11:**
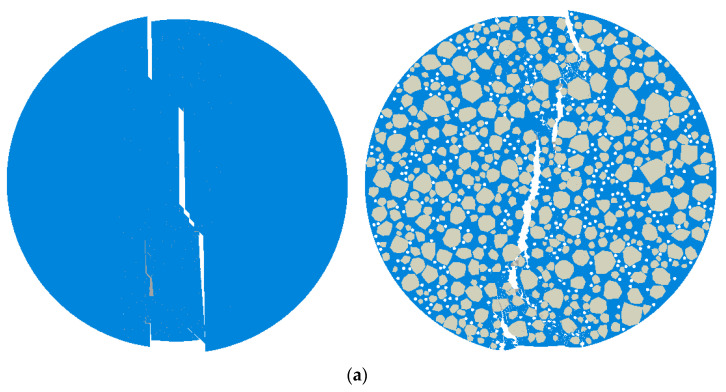

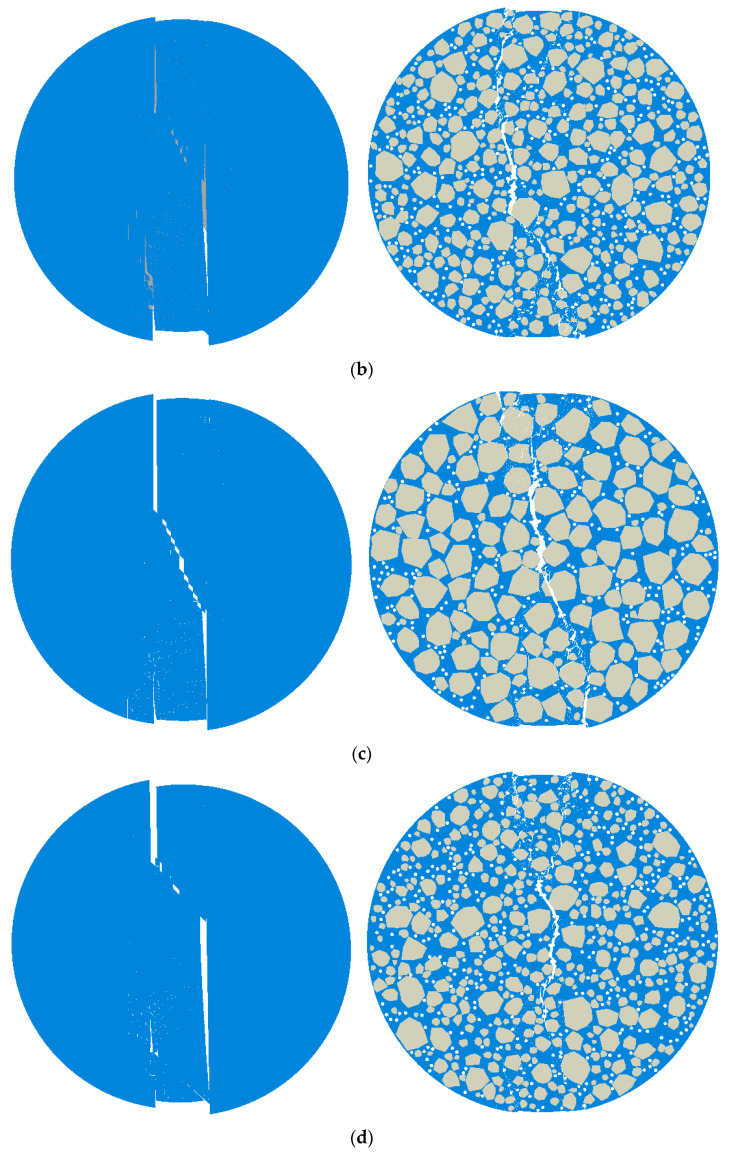

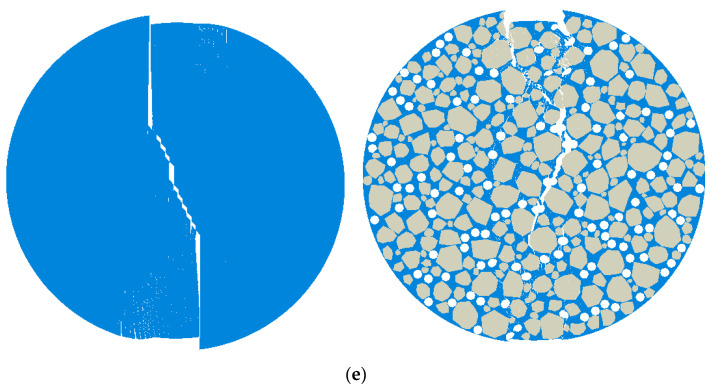
Deformation contour plot: (**a**)Mix-1, (**b**) Mix-2, (**c**) Mix-3, (**d**) Mix-4, and (**e**) Mix-5.

**Figure 12:**
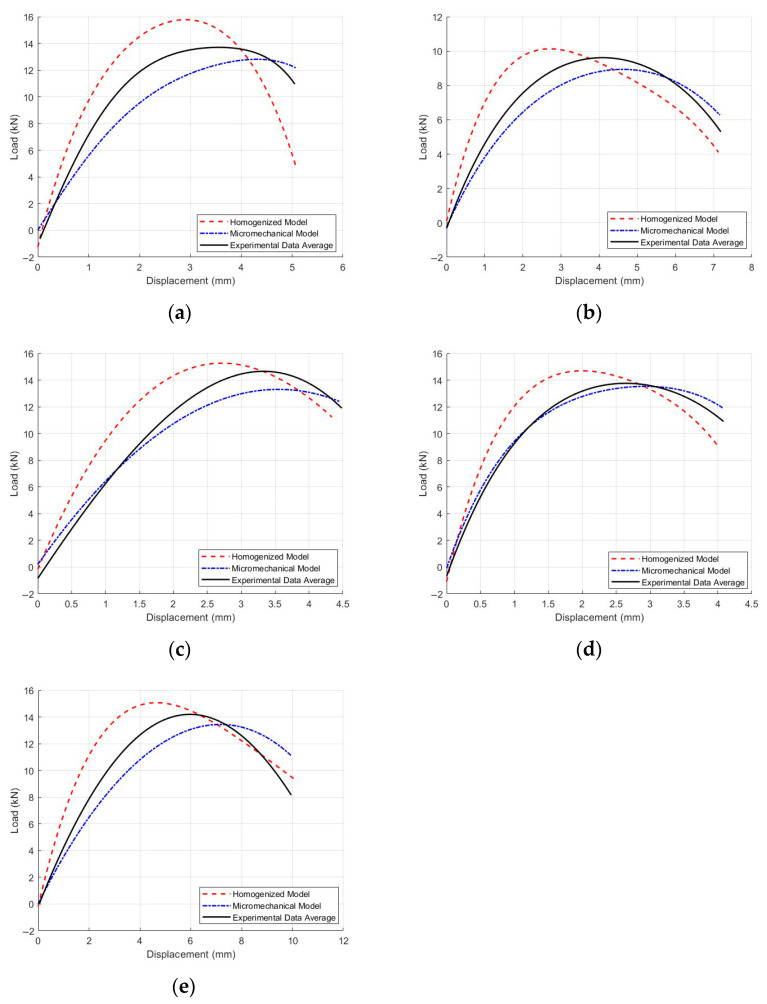
Load–displacement curves of the IDT FE simulation for five asphalt mixtures: (**a**) Mix-1, (**b**) Mix-2, (**c**) Mix-3, (**d**) Mix-4, and (**e**) Mix-5.

A homogenized model and a mesoscopic model for five asphalt mixtures were established in ABAQUS. The software was used to compute the load–displacement curves for each model and the deformation contour maps, as shown in Figure 11 (gray: coarse aggregates; blue: asphalt mastic). These results were then compared with the average load–displacement curves calculated from the three experimental samples for each material, as illustrated in Figure 12.

### 4.2. Quantitative Evaluation Metrics and Error Analysis

#### 4.2.1. Statistical Metrics

The coefficient of determination (R^2^) was used as a crucial statistical indicator to evaluate the accuracy of the FEM. The R^2^ value ranges from 0 to 1, with values closer to 1 indicating a higher degree of fit between the model and the actual data. In this study, by calculating the R^2^ values of the homogenized model and the mesoscopic model, as shown in Table 6, a direct comparison was made to assess which model more accurately reflects the load–displacement response of the IDT. Specifically, if the R^2^ value of the mesoscopic model was significantly higher than that of the homogenized model, it indicated that the mesoscopic model had a greater advantage in simulating the material’s internal heterogeneity and localized stress concentration.

To further interpret the agreement between the simulation results and the experimental load–displacement curves, Pearson’s correlation coefficient was computed for each mixture type. The Pearson coefficient (r) ranges from –1 to 1, where values closer to 1 indicate a strong positive linear relationship. In this study, the r-values between the micromechanical simulation results and experimental data ranged from 0.94 to 0.99, indicating very strong correlation and confirming the high predictive capability of the micromechanical model.

In contrast, the Pearson correlation coefficients for the homogeneous model ranged from 0.78 to 0.88, reflecting moderate-to-strong correlation, but with lower consistency than the micromechanical approach. These statistical outcomes were consistent with the interpretation methods suggested by Decky et al. [29] in evaluating correlation strength for pavement analysis models.

#### 4.2.2. Mesh Sensitivity Analysis

**Figure 13:**
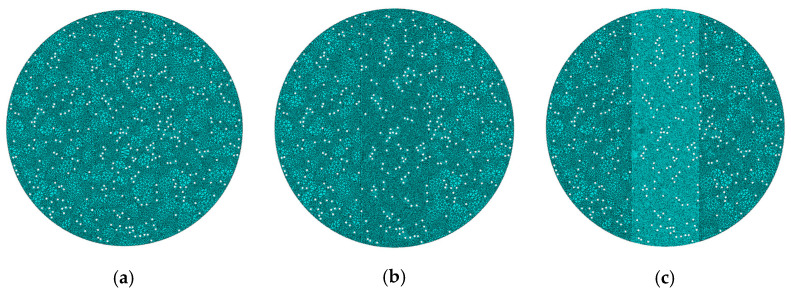
Mesh division schemes of the Mix-1 micromechanical model with different element sizes for the mesh sensitivity analysis: (**a**) 2 mm, (**b**) 1 mm, and (**c**) 0.5 mm.

**Figure 14:**
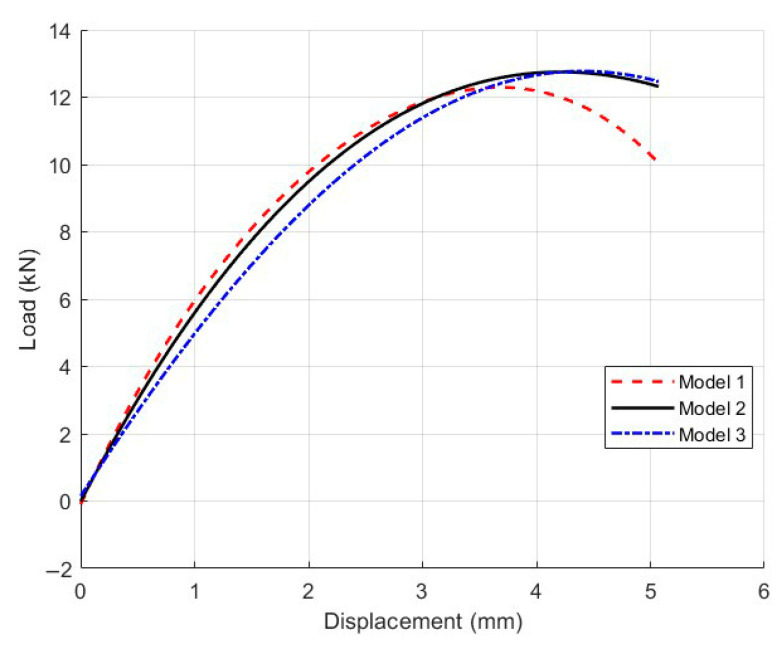
Load–displacement curves of the Mix-1 micromechanical model with different mesh sizes for the mesh sensitivity analysis.

To verify the mesh independence of the micromechanical model, a mesh sensitivity analysis was conducted using the Mix-1 specimen. Three models with different mesh sizes in the central region were developed: Model 1 (2 mm), Model 2 (1 mm), and Model 3 (0.5 mm), shown in Figure 13. The light-colored appearance in some areas is due to the finer mesh elements themselves, not a deliberate color coding. This reflects the higher mesh density in those regions of the finite element model. The corresponding load–displacement curves are presented in Figure 14.

The results showed that the peak load of Model 3 (12.30 kN) was only 0.9% higher than that of Model 2 (12.20 kN), while Model 1 yields a visibly lower peak value of 11.80 kN. Moreover, the curve shapes of Model 2 and Model 3 closely matched each other throughout the loading process. These findings indicated that the 1 mm mesh provided sufficient accuracy while maintaining computational efficiency [30]. Therefore, all subsequent micromechanical simulations in this study employed the 1 mm mesh size.

#### 4.2.3. Discussion on Error Sources

In numerical simulations, errors may arise from several sources:

- Differences in material characterization approaches: The homogeneous model simplifies asphalt mixtures as macroscopically equivalent continuous media, neglecting the distribution and interfacial characteristics of actual constituents, including coarse aggregates, asphalt mortar, and voids. Although this simplification enhances computational efficiency, it inadequately represents the effects of material heterogeneity on crack propagation paths and localized stress concentrations.
- Precision in interface behavior modeling: The mesoscale model achieves superior prediction accuracy through explicit implementation of cohesive elements at aggregate–mortar interfaces and within mortar phases, enabling the precise simulation of interface debonding, micro-crack initiation, and damage evolution processes. In contrast, while CZMs are also employed in homogeneous approaches, their equivalent distribution mechanism fails to capture the preferential interfacial crack propagation observed in actual materials, resulting in deviations from experimental measurements.
- Structural scale and crack evolution paths: The mesoscale framework effectively simulates crack deflection mechanisms influenced by coarse aggregate gradation, spatial distribution, and skeletal structure, thereby accurately reproducing nonlinear phases and fracture processes in experimental curves. Homogeneous models, however, disregard these microstructural mechanisms, exhibiting accelerated stiffness degradation and load reduction during crack propagation stages.

In summary, fundamental discrepancies between homogeneous and mesoscale models in material characterization, interface modeling fidelity, fracture evolution mechanisms, and parameter selection constitute the primary causes of deviations in load–displacement curve predictions. Consequently, meso-structure based modeling strategies demonstrate greater potential and superiority for engineering applications requiring high prediction accuracy or material design optimization studies.

## 5. Application of Mesoscopic Models

In the previous section, it was concluded that the mesoscopic model, due to its ability to accurately account for the interactions among the coarse aggregates, asphalt mastic, and voids, demonstrates higher reliability and accuracy in predicting the actual mechanical response compared to the homogeneous model. Thus, the mesoscopic model of Mix-1 was used as a representative to further investigate the inappropriate compaction level, gradation design, and mixing effort on the mechanical behavior of asphalt mixtures. In each discussion, Model 2 represented the original mesoscopic model of Mix-1.

### 5.1. Influence of Incorrect Compaction Level on Mechanical Response of Mesoscopic Models

#### 5.1.1. Model Parameters and Computational Results

**Figure 15:**
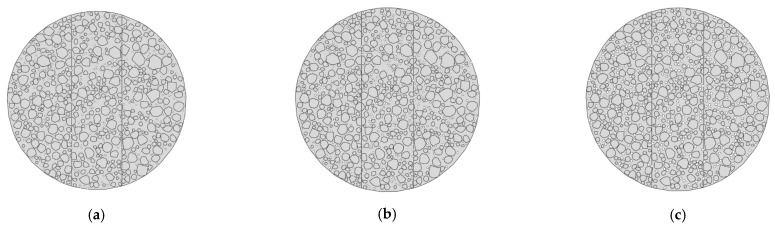
Three mesoscopic models with different air void contents for Mix-1: (**a**) Model 1, (**b**) Model 2, and (**c**) Model 3.

**Figure 16:**
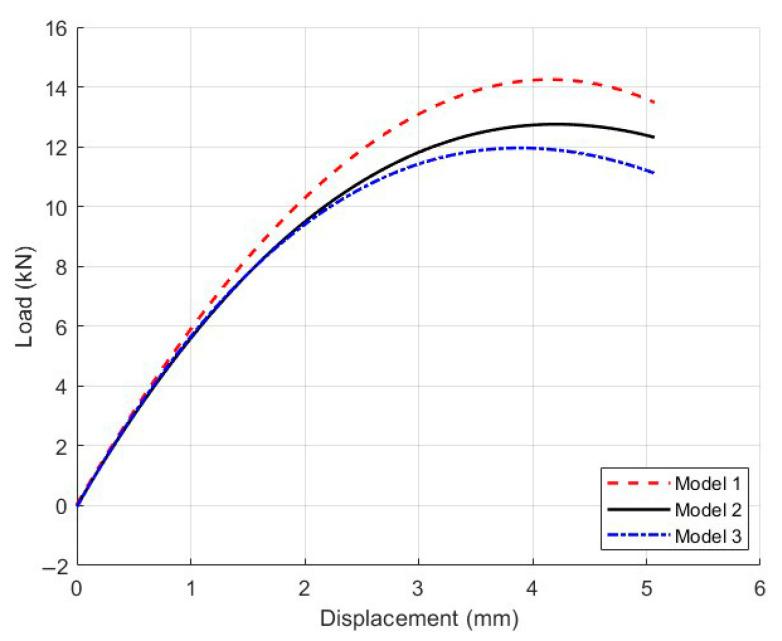
Load–displacement curves of the mesoscopic models for Mix-1 with three air void contents.

**Figure 17:**
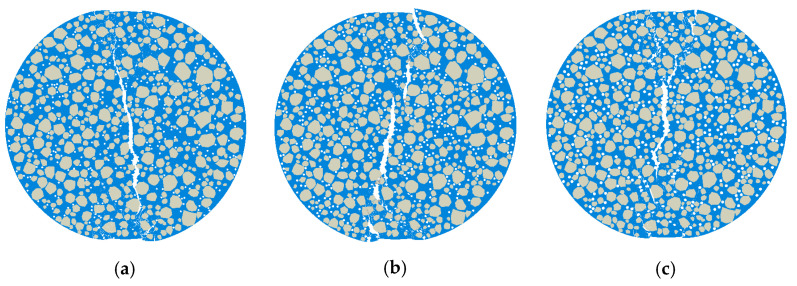
Deformation contour plot: (**a**) Model 1, (**b**) Model 2, and (**c**) Model 3.

The microscopic modeling process of Mix-1 is detailed in Section 3.3. Under the premise of maintaining the gradation and distribution of coarse aggregates, models with air void content of 4% (model 1), 7% (model 2), and 10% (model 3) were established as shown in Figure 15. The corresponding average pore radius for each porosity are provided in Table 7. The load–displacement curves for each model are calculated and presented in Figure 16, while the deformation contour plots are displayed in Figure 17.

#### 5.1.2. Data Comparison and Analysis of Influencing Factors

Figure 16 illustrates the load–displacement responses of the models with varying air void contents. Overall, as the air void content increased from 4% to 10%, a noticeable reduction in peak load was observed, accompanied by a continuous decline in stiffness. Model 1, with the lowest void ratio (4%), exhibited superior compressive resistance and structural rigidity. It demonstrated that for the highest peak load, the initial slope of the curve was significantly steeper, indicating higher stress was required during the early deformation stage. In contrast, Model 3 (10% voids) presented a considerable drop in both initial stiffness and peak load. Moreover, the post-peak softening was more pronounced, suggesting a substantial degradation in load-bearing capacity and structural integrity due to increased void content.

The deformation contour plots in Figure 17 further revealed how varying void ratios influence the failure mechanisms. In Model 1, damage localized near the loading axis, and the crack propagated stably along the direction of the applied load. The fracture zone remained narrow, indicative of a typical brittle failure pattern. Conversely, Model 3 showed widespread strain distribution and a more scattered damage zone, with cracks propagating in multiple directions, reflecting a ductile failure behavior.

From the perspective of crack propagation, specimens with lower air voids tend to develop cracks that align with the principal stress direction, characterized by short and rapid crack paths and abrupt failure. At higher void contents, internal voids disrupted the stress transmission network, forcing cracks to navigate around these discontinuities. This resulted in more tortuous propagation paths, earlier crack initiation, slower crack growth, and a wider damage region. Therefore, insufficient compaction severely compromises the structural stability of asphalt mixtures, leading to a significant decline in cracking resistance, and should be considered a critical quality control factor during field construction.

### 5.2. Influence of Gradation Design Classification on Mechanical Response of Mesoscopic Models

#### 5.2.1. Model Parameters and Computational Results

**Figure 18:**
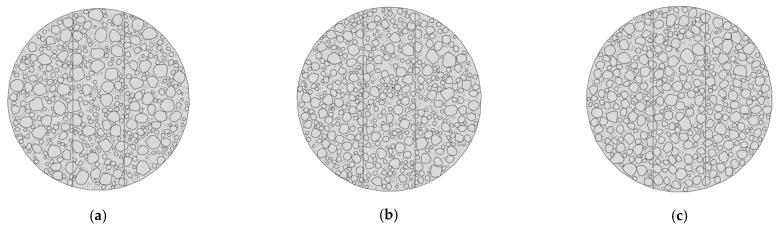
Three different gradation-based mesoscopic models of Mix-1: (**a**) Model 1, (**b**) Model 2, and (**c**) Model 3.

**Figure 19:**
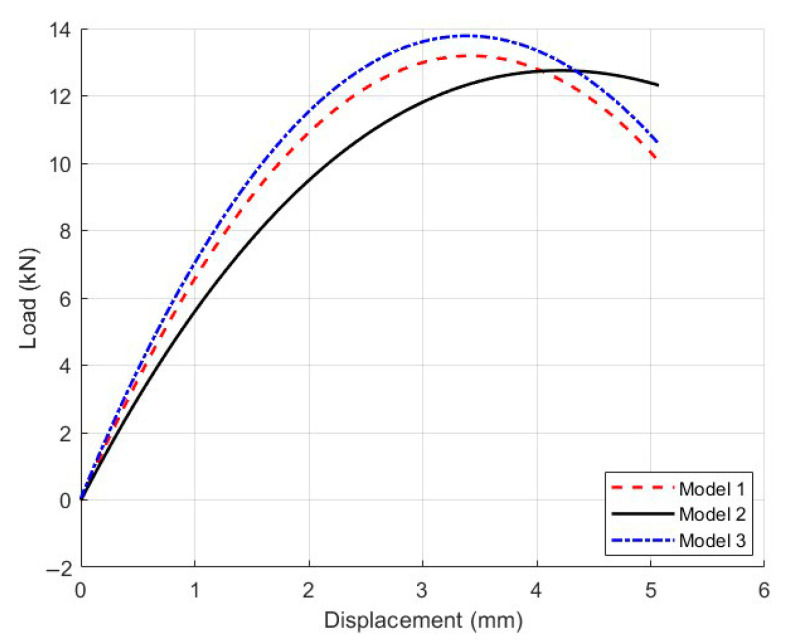
Load–displacement curves of three different gradation-based mesoscopic models of Mix-1.

**Figure 20:**
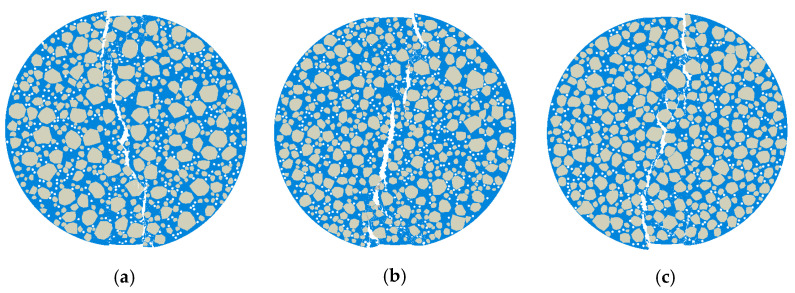
Deformation contour plot: (**a**) Model 1, (**b**) Model 2, and (**c**) Model 3.

For the microscopic modeling of Mix-1, the coarse aggregates were typically modeled in strict accordance with the gradation, established step by step. With the porosity maintained at 7% and the total content of coarse and fine aggregates unchanged, the coarse aggregate gradation was modeled according to Table 8. The corresponding microscopic model is shown in Figure 18, and the load–displacement curves for each model are shown in Figure 19. The deformation contour plots are illustrated in Figure 20.

#### 5.2.2. Data Comparison and Analysis of Influencing Factors

Figure 19 compares the load–displacement behavior of models designed with different aggregate gradations. Model 2, utilizing a continuously graded aggregate distribution, demonstrates superior performance with the highest peak load and initial stiffness among the three. The even distribution of aggregate sizes forms a robust interlocking structure, enhancing the material’s load-bearing capacity and overall structural integrity. In contrast, Model 1 and Model 3 lack specific aggregate sizes—9.5 mm and 4.75 mm, respectively—resulting in discontinuous gradation. This gap-graded configuration weakens interparticle contact and disrupts the structural support system, leading to significantly reduced mechanical properties, including both peak strength and ductility.

As depicted in the strain contour plots in Figure 20, different gradation schemes influence both damage localization and crack development. Model 2 features a narrow zone of concentrated strain, with cracks propagating clearly along the primary loading axis, indicating a controlled failure mode. However, the absence of key aggregate sizes in Model 1 and Model 3 introduces unsupported regions within the internal structure, causing localized stress concentrations. Consequently, multiple cracks initiate and propagate in divergent directions, expanding the damage zone and compromising structural continuity.

The crack propagation patterns further indicate that well-designed gradation can form a continuous skeleton structure at the microscale. This not only supports the asphalt mortar effectively but also improves stress distribution and suppresses crack growth. In contrast, gap-graded systems exhibit disrupted load paths and intensified local stress concentrations, facilitating crack initiation and propagation. These findings underscore the critical importance of aggregate gradation design in ensuring the cracking resistance of asphalt mixtures during the mix design phase.

### 5.3. Influence of Mixing Effort on Mechanical Response of Mesoscopic Models

#### 5.3.1. Model Parameters and Computational Results

**Figure 21:**
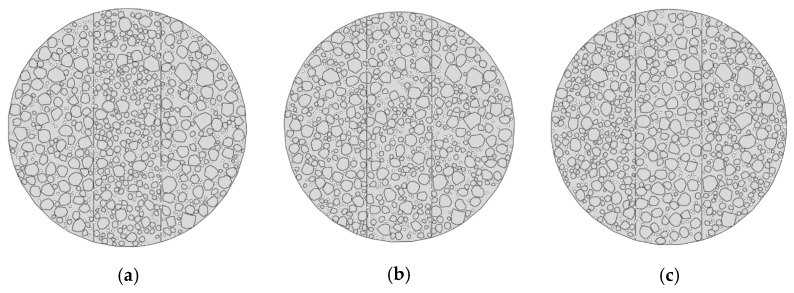
Three different coarse aggregate distribution mesoscopic models for Mix-1: (**a**) Model 1, (**b**) Model 2, and (**c**) Model 3.

**Figure 22:**
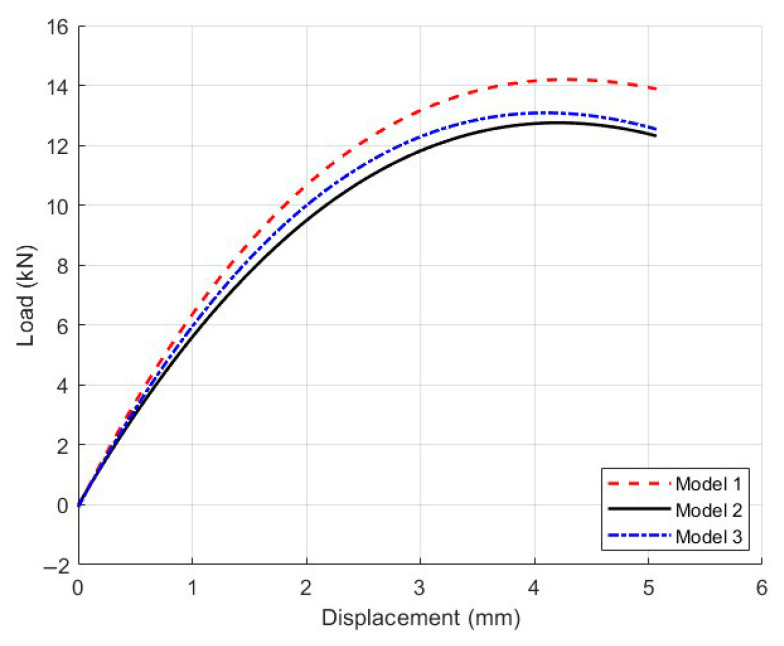
Load–displacement curves of three different coarse aggregate distribution mesoscopic models for Mix-1.

**Figure 23:**
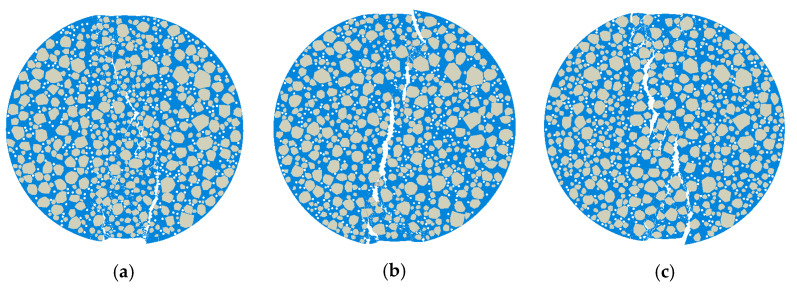
Deformation contour plot: (**a**) Model 1, (**b**) Model 2, and (**c**) Model 3.

In the microscopic modeling of Mix-1, the coarse aggregates were assumed to be evenly distributed within the model. However, in the actual specimen preparation process, uniform mixing effort often leads to localized concentrations of coarse aggregates. Under the premise of modeling the coarse aggregates according to the gradation, the distribution of 9.5 mm–2.36 mm coarse aggregates was adjusted to be concentrated in the center and edge regions, with the respective area percentages modeled as shown in Table 9. The microscopic model for this case is shown in Figure 21, the load–displacement curves for each model are presented in Figure 22, and the deformation contour plots are displayed in Figure 23.

#### 5.3.2. Data Comparison and Analysis of Influencing Factors

As shown in Figure 22, variations in aggregate distribution caused by different mixing uniformities had a pronounced effect on mechanical response at the microscale. In Model 2, coarse aggregates were evenly dispersed, leading to uniform stress distribution across the specimen. This resulted in the highest peak load and optimal ductility. On the contrary, Model 1 and Model 3 exhibited aggregate clustering either at the center or near the boundary, causing localized densification of the skeleton structure and uneven stress transmission pathways, which in turn reduce both the overall stiffness and load-carrying capacity.

Figure 23 presents the deformation contour plots, which further clarify the failure modes associated with different mixing qualities. Model 2 exhibited a centralized crack that propagated cleanly along the main loading direction, indicating effective damage control. In contrast, Model 1 suffered from excessive central aggregate concentration, making the core region a critical stress concentration point and leading to premature failure. Meanwhile, Model 3 showed aggregate accumulation near the edge, triggering high peripheral stress and resulting in cracks that initiated at the boundary and propagate asymmetrically toward the center, forming an irregular failure pattern.

Crack development analysis revealed that non-uniform aggregate distribution disrupted internal stress equilibrium, generating localized stress concentrations that serve as preferential crack initiation sites. Moreover, aggregate clustering restricted the even dispersion of asphalt mortar, undermining the cohesion at the binder–aggregate interface and accelerating material degradation. These observations highlighted that proper control of the mixing process is essential in real-world construction. Uniform mixing directly governed the microscale structural integrity and significantly impacted the cracking performance of asphalt mixtures.

## 6. Conclusions

This study aimed to investigate the cracking performance of asphalt mixtures by comparing homogenized and three-phase micromechanical finite element models based on the cohesive zone model (CZM) and to further evaluate the influence of construction-related defects on the fracture behavior change.

- The micromechanical model achieved significantly better agreement with experimental IDT results, with R^2^ values ranging from 0.86 to 0.98, compared to 0.66 to 0.77 for the homogenized model.
- It accurately captured crack initiation and propagation paths and better reflected localized damage behavior.
- Simulation of construction deficiencies revealed strong sensitivity in the fracture resistance.
- Increasing air void content from 4% to 10% led to a peak load reduction of up to 35% and stress concentration increases of 25–40%.
- Gap-graded structures accelerated crack coalescence and reduced peak load by 12.5–19.3%.
- In contrast, a dense and uniform aggregate distribution enhanced stress transfer efficiency and improved peak load capacity by 15.4% while also delaying crack propagation.

Compared to existing micromechanical modeling studies, the novelty of this work lies in its dual focus on both methodological refinement and practical application. By integrating a stochastic aggregate generation algorithm, three-phase material representation, and parameter calibration, the micromechanical model achieves high accuracy in simulating IDT fracture behavior. More importantly, this study pioneers the quantitative evaluation of typical field construction issues—such as insufficient compaction and poor mixing—on cracking resistance using micromechanical modeling. These innovations extend the application boundary of FE-based modeling and offer practical insights for pavement design and maintenance.

However, to address the existing limitations and uncertainties in the current work, future research is need to primarily focus on the following three directions:

In the finite element (FE) simulation of asphalt mixture indirect tensile tests (IDTs), the rational setting of boundary conditions proved crucial for ensuring the accuracy of results. Loading methods and support constraints were required to replicate experimental conditions closely, with appropriate tangential friction coefficients applied at contact interfaces to avoid stress distribution distortion and deviations in the load–displacement response. Following this, sensitivity analyses were recommended based on experimental data or the established literature. In terms of mesh discretization, the quality of meshing significantly influenced the model’s capability to capture stress concentrations, especially at material interfaces where stiffness discontinuities occurred. Coarse meshes often failed to reflect micro-crack initiation and propagation paths, while excessively refined meshes, though improving precision, greatly increased computational costs and solution time. In explicit dynamic analyses, it was necessary to balance time increments with mesh density and material properties to prevent response lag and convergence difficulties. Furthermore, model calibration was advanced by integrating Abaqus and Isight (Version 2021, Dassault Systèmes, France)allowing for the automatic execution of parameter modification, computation, and result extraction. By applying sensitivity analyses and multi-objective optimization algorithms such as genetic algorithms, material parameters were iteratively updated to minimize the root mean square error of the load–displacement curve, thus establishing a reliable foundation for simulating crack initiation and fatigue failure in asphalt pavements.

## Figures and Tables

**Table 1:** Design properties of asphalt mixture.

Asphalt Mixture Designation	Mix-1	Mix-2	Mix-3	Mix-4	Mix-5
Asphalt Content (%)	5.2	4.6	6.0	5.1	6.7
RAP Content (%)	20.1	30.0	12.1	20.0	0
Air Voids (%)	7.14	6.65	7.45	7.20	19.2

**Table 2:** Aggregate gradation of asphalt mixture.

Asphalt Mixture Designation	Sieve Size (mm)	19–16	16–9.5	9.5–4.75	4.75–2.36	2.36–0
Mix-1	Percent (%)	0	7.5	35.5	21.8	29.4
Mix-2	Percent (%)	7.0	17.0	26.2	18.0	28.0
Mix-3	Percent (%)	0.7	76.7	7.5	4.3	7.1
Mix-4	Percent (%)	0	12.8	26.5	21.6	33.4
Mix-5	Percent (%)	0	42.0	38.9	12.8	5.1

**Table 3:** Material properties for the homogeneous model.

Material Parameters		Density (kg/m^3^)	Poisson’s Ratio	Elastic Modulus (MPa)	Cohesive Element Parameters		
					Interface Stiffness K (MPa/mm)	Tensile Strength T_C_ (MPa)	Fracture Energy G_C_ (J/m^2^)
Asphalt Mixture Designation	Mix-1	2400	0.35	1296	480	1.9	1850
	Mix-2	2400	0.35	1651	611	1.18	1890
	Mix-3	2300	0.35	4543	1628	2.03	2590
	Mix-4	2400	0.35	2670	988	1.86	2190
	Mix-5	2200	0.35	1434	531	1.79	3260

**Table 4:** Void size distribution of asphalt mixture.

Asphalt Mixture Designation	Mix-1	Mix-2	Mix-3	Mix-4	Mix-5
Air Voids (%)	7.14	6.65	7.45	7.20	19.2
Air Voids Radius (mm)	0.80	0.77	0.82	0.80	2.06

**Table 5:** Micromechanical model material parameters.

Material Parameters		Material	Elastic Modulus (MPa)	Poisson’s Ratio	Density (kg/m^3^)	Cohesive Element Parameters		
						Interface Stiffness K (MPa/mm)	Tensile Strength T_C_ (MPa)	Fracture Energy G_C_ (J/m^2^)
Asphalt Mixture Designation	Mix-1	Aggregate	55,000	0.15	2500	55,000	6.35	1250
		Mortar	518	0.35	2200	192	1.32	1600
		Interface	518	0.35	2200	192	1.19	800
	Mix-2	Aggregate	55,000	0.15	2500	55,000	6.35	1250
		Mortar	660	0.35	2200	245	0.83	1510
		Interface	660	0.35	2200	245	0.75	760
	Mix-3	Aggregate	55,000	0.15	2500	55,000	6.35	1250
		Mortar	1817	0.35	2200	673	1.42	2070
		Interface	1817	0.35	2200	673	1.28	1040
	Mix-4	Aggregate	55,000	0.15	2500	55,000	6.35	1250
		Mortar	1068	0.35	2200	395	1.3	2070
		Interface	1068	0.35	2200	395	1.17	1040
	Mix-5	Aggregate	55,000	0.15	2500	55,000	6.35	1250
		Mortar	573	0.35	2200	212	1.25	2890
		Interface	573	0.35	2200	212	1.13	1450

**Table 6:** R^2^ for homogeneous model and micromechanical model.

Asphalt Mixture Designation	Model Type	R^2^
Mix-1	Homogenized Model	0.6972
	Micromechanical Model	0.9083
Mix-2	Homogenized Model	0.6635
	Micromechanical Model	0.9134
Mix-3	Homogenized Model	0.7728
	Micromechanical Model	0.9612
Mix-4	Homogenized Model	0.7367
	Micromechanical Model	0.9875
Mix-5	Homogenized Model	0.7755
	Micromechanical Model	0.8617

**Table 7:** Microstructural void sizes for different porosities in Mix-1.

Model Name	Air Voids (%)	Air Voids Radius (mm)
Model 1	4	0.65
Model 2	7	0.79
Model 3	10	1.00

**Table 8:** Gradation of different mixes for Mix-1.

Model Name	Sieve Size (mm)	16	13.2	9.5	4.75	2.36
Model 1	Percent Passing (%)	100	99.2	-	57	35.2
Model 2	Percent Passing (%)	100	99.2	92.6	57	35.2
Model 3	Percent Passing (%)	100	99.2	92.6	-	35.2

**Table 9:** Distribution of coarse aggregates in Mix-1.

Model Name	Region	Coarse Aggregate Size Groups (mm) and Proportions (%)	
		9.5–4.75	4.75–2.36
Model 1	Center	15.79	37.49
	Edge	32.11	10.41
Model 2	Center	29.67	18.22
	Edge	29.67	18.22
Model 3	Center	43.57	4.34
	Edge	21.87	26.04

## Data Availability

The original contributions presented in the study are included in the article; further inquiries can be directed to the corresponding author.
